# Digital versus conventional surgical guide fabrication: A randomized crossover study on operator preference, difficulty, effectiveness, and operating time

**DOI:** 10.1002/cre2.831

**Published:** 2024-01-18

**Authors:** Vincent J. J. Donker, Karel H. Heijs, Christiaan W. P. Pol, Henny J. A. Meijer

**Affiliations:** ^1^ Department of Oral and Maxillofacial Surgery, University of Groningen University Medical Center Groningen Groningen The Netherlands; ^2^ Dental School, University of Groningen University Medical Center Groningen Groningen The Netherlands; ^3^ Department of Restorative Dentistry, University of Groningen University Medical Center Groningen Groningen The Netherlands

**Keywords:** dental education, digital workflow, operator preference, surgical guide fabrication

## Abstract

**Aim:**

If surgical guide fabrication is introduced in a dental education program, a digital and conventional workflow can be used. This study evaluated operator preference, perceived difficulty and effectiveness and operating time of both fabrication methods.

**Materials and Methods:**

Forty participants in a university setting (students, *n* = 20; dentists, *n* = 20) with varying levels of dental experience, but no experience in surgical guide fabrication, were randomly assigned to consecutively fabricate surgical guides on a standardized training model, with either the digital or conventional workflow first. The operating time was measured, and operator preference and the perception of difficulty and effectiveness were assessed with a questionnaire. T tests were used for statistical analysis (α = .05).

**Result:**

Of the students, 95% preferred the digital workflow and of the dentists 70%. The perceived difficulty of the digital workflow was significantly lower than the conventional workflow in the student group. Both groups perceived the digital workflow to be more effective. The mean operating time (mm:ss) amounted 12:34 ± 2:24 (students) and 18:07 ± 6:03 (dentists) for the digital, and 22:20 ± 3:59 (students) and 20:16 ± 4:03 (dentists) for the conventional workflow.

**Conclusion:**

Both students and dentists prefer the digital workflow for surgical guide fabrication. Students perceive the digital workflow as less difficult and more effective than the conventional workflow. The operating time for surgical guide fabrication is shorter with a digital workflow. This study indicates that digital fabrication techniques for surgical guides are preferred to be incorporated into the dental curriculum to teach students about treatment planning in implant dentistry.

## 1 INTRODUCTION

The focus of investigation in implant dentistry has shifted from implant survival to implant placement (Varga et al., 2019).

Implant placement is considered to be optimal when it is planned prosthetically driven, taking into account the surrounding anatomical structures (Katsoulis et al., 2009; Tahmaseb et al., 2018). Placing an implant according to the planned position can be facilitated by means of guided implant surgery, that is, by using a surgical guide that facilitates the transfer of the planned implant position to the patient. Surgical guides are considered effective and accurate (Younes et al., 2019). Studies on the accuracy of implant placement report greater accuracy with respect to the planned position of the implant when surgical guides were used compared to freehand implant surgery (Tahmaseb et al., 2014, 2018; Van Assche et al., 2012).

A surgical guide can be manufactured with either a digital or a conventional workflow. A conventional surgical guide is made of acrylic resin and requires a stone cast and a wax set‐up (Chiche et al., 1989; Neidlinger et al., 1993). Digital fabrication of a surgical guide with computer‐aided design (CAD) requires an intraoral scan and often a cone beam computed tomography (CBCT) scan of the intended implant site (Neugebauer et al., 2011; Whitley et al., 2017). The CAD surgical guide can then be produced with computer‐aided manufacturing (CAM), either by milling or printing. Digitally and conventionally manufactured surgical guides have been compared for accuracy, and digitally manufactured surgical guides have been shown to be more reliable (Farley et al., 2013; Reyes et al., 2015). In addition to accuracy, operator preference and operating time can be studied to compare the digital and conventional workflows. Apart from the overall preference towards a method, the perception of difficulty and effectiveness also influence the willingness of those involved to incorporate a new treatment modality into practice or in the dental curriculum (Kirchner et al., 2020). The perceived difficulty of a method is determined by how difficult the steps involved feel and whether the operator feels confident of achieving a consistent end result. Perceived effectiveness is determined by whether the operator feels that the method is capable of producing the end result in a logical and timely manner (Joda et al., 2017).

In a comparison of making a digital and a conventional impression of a dental training model, both dental students and experienced clinicians preferred the digital method in terms of difficulty and effectiveness, and the operating time for making a digital impression was shorter than that for making a conventional impression (Joda et al., 2017; Lee & Gallucci, 2013; Lee et al., 2013; Zitzmann et al., 2017). There is currently no evidence on these factors comparing the manufacture of surgical guides. Therefore, the purpose of this study was to evaluate the digital and conventional fabrication of surgical guides with respect to operator preference and perception of difficulty and effectiveness and operating time.

The null hypotheses were: (1) there is no difference in perceived difficulty between digital and conventional fabrication of surgical guides; (2) there is no difference in perceived effectiveness between digital and conventional fabrication of surgical guides; and (3) there is no difference in required operating time between digital and conventional fabrication of surgical guides.

## 2 MATERIALS AND METHODS

### 2.1 Study design

In this randomized crossover study 40 participants with varying levels of dental experience were enrolled to evaluate a digital and conventional fabrication method of surgical guides. The research took place in the Dental School of the University Medical Center Groningen (UMCG), the Netherlands. The study protocol was reviewed by the Medical Ethics Review Board of the UMCG (METc 2021/454). Written informed consent was obtained from the participants before enrollment. This manuscript follows the CONSORT guidelines for randomized crossover studies (Dwan et al., 2019; Schulz et al., 2010).

### 2.2 Participants

The participants consisted of two groups: dental students without any clinical experience (*students*) and qualified dentists in a university setting with at least 1 year of clinical experience (*dentists*). The students and dentists had no prior experience in surgical guide fabrication. The participants were randomly assigned (computer generated random number) to consecutively fabricate surgical guides, in which either the digital or conventional fabrication method was used first (crossover design with counterbalancing to reduce the carryover effect). All participants were initially instructed through a step‐by‐step tutorial video for both manufacturing methods.

### 2.3 Standardized model

A digital scan (TRIOS 3, 3Shape, Copenhagen, Denmark) and a type 4 dental stone cast (Fujirock EP, GC Europe, Leuven, Belgium) of a standardized dental training model with a missing mandibular left first molar were used. A digital set‐up was designed with dental design software (CARES Visual, Version 13.0, Institut Straumann AG, Basel, Switzerland) and a wax set‐up (Thowax, Yeti Dentalprodukte GmbH, Engen, Germany) was made on the stone cast. The stone cast was duplicated 40 times in a silicon mold (Heraform, Kulzer, Hanau, Germany) as well as the wax set‐up with a silicon index (Provil novo, Kulzer, Hanau, Germany).

### 2.4 Digital workflow

**Figure 1:**
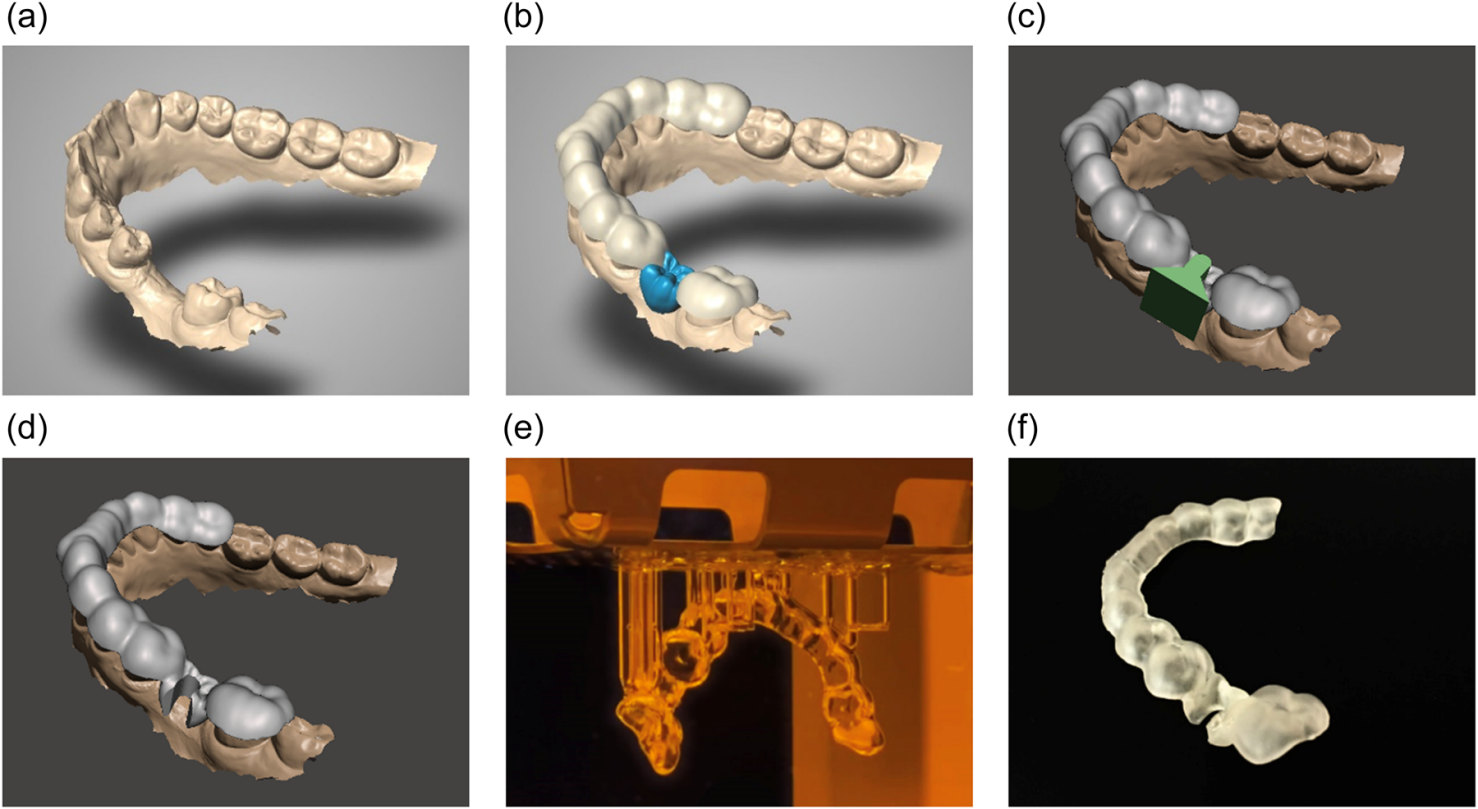
(a–f) Digital workflow. (a) The digital scan is loaded into the dental design software (CARES visual). (b) The surgical guide is generated according to the defined outline and the digital set‐up. (c) The files are exported to another software (MeshMixer) and a three‐dimensional shape (in green) is used to determine the position of the drill hole. (d) By using Boolean difference, the drill hole is digitally trimmed from the surgical guide. (e) The surgical guide is manufactured with a 3D printer. (f) Excess print material is removed, and the surgical guide is washed in ethanol.

The digital workflow consisted of loading the digital scan and the set‐up into the dental design software (CARES Visual) and defining the outline for the surgical guide. The digital outline had to extend from the mandibular left second molar to the mandibular right second premolar and had to be placed above the undercuts on the digital scan. The file of the surgical guide was then transferred to another software (MeshMixer, Version 3.5, Autodesk, San Rafael, CA, United States) to create the drill hole. The position of the drill hole was determined by means of a three‐dimensional shape in the diameter of a pilot‐drill and then trimmed from the surgical guide (Boolean difference). Subsequently, the file of the surgical guide with drill hole was exported to a 3D printer (NextDent 5100, 3D Systems, Rock Hill, SC, United States) and printed with resin (NextDent SG, 3D Systems). The sequence of the digital workflow is summarized in Table 1 and illustrated in Figure 1a–f.

**Table 1:** Sequence of the digital workflow.

Workflow steps	Procedures
Defining the outline	1. Loading the digital scan and the digital set‐up into the design software (CARES Visual). 2. Defining the outline on the digital scan. 3. Export of the surgical guide file.
Determining the drill hole position	4. Loading the surgical guide file into another software (MeshMixer). 5. Defining the position of drill hole and trimming the surgical guide. 6. Export of the surgical guide file.
Printing of the surgical guide	7. Loading the surgical guide file in printer. 8. Printing of the surgical guide.

### 2.5 Conventional workflow

**Figure 2:**
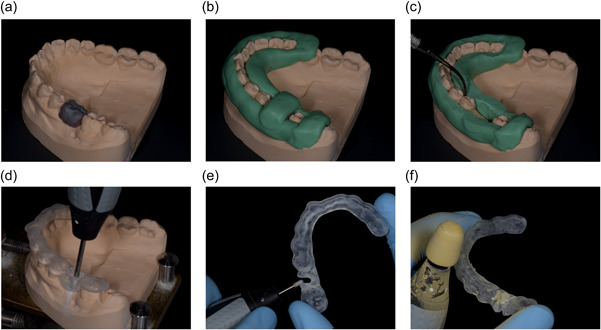
(a–f) Conventional workflow. (a) Stone cast with the wax set‐up. (b) A silicon putty mold is created on the stone cast. (c) The putty mold is adjusted with a scalpel, the wax set‐up is removed, and undercuts and interdental embrasures are covered with wax. (d) The drill hole is made with a drilling machine mounted on a surveyor. (e) The drill hole is adjusted to allow access from the buccal side. (f) The surgical guide is polished with a polishing rubber.

The conventional workflow consisted of making the outline for the surgical guide on the stone cast with silicon putty (Provil novo). The putty mold had to extend from the mandibular left second molar to the mandibular right second premolar and had to be above undercuts on the stone cast. Where necessary, the height of the putty mold was adjusted with a scalpel (No. 15, Swann Morton, Sheffield, United Kingdom). The wax set‐up was then removed from the stone cast and undercuts and interdental embrasures were covered with wax (Yellow Wax, Cavex, Haarlem, the Netherlands) with an instrument (P.K. Thomas N.2, Chicago, IL, United States) heated by induction (Noflame Plus, Amann Girrbach AG, Koblach, Austria). The stone cast was covered with separating liquid (ISO‐K, Candulor, Opfikon, Switzerland) and transparent polymethyl methacrylate (PMMA) (AutoPlast, Candulor) was poured onto the stone cast and putty mold to make the surgical guide. Polymerization took place in a pressure vessel for 15 min at a temperature of 40°C and a pressure of 2 bar, in accordance with the manufacturer's instructions. After polymerization, the position of the drill hole was determined with the surgical guide on the stone cast. A hole with the diameter of the pilot drill was made in the surgical guide with a drilling machine mounted on a surveyor. The hole was to be positioned in the center of the fissure of the set‐up and of the alveolar process on the stone cast. The drill hole and surgical guide were then finished with a handpiece with rotating instruments and a polishing rubber (H23RSE, H251EQ, 9641, Komet Dental, Lemgo, Germany). The sequence of the conventional workflow is summarized in Table 2 and illustrated in Figure 2a–f.

**Table 2:** Sequence of the conventional workflow.

Workflow steps	Procedures
Making outline and blocking undercuts	1. Making a silicon putty mold on stone cast. 2. Adjustment of the putty mold. 3. Removal of wax set‐up from stone cast and covering undercuts and interdental embrasures with wax.
Pouring and setting of the PMMA	4. Covering stone cast with separating liquid. 5. Pouring PMMA on stone cast and putty mold. 6. Polymerization in a pressure vessel.
Making the drill hole and finishing	7. Making the drill hole with a drilling machine mounted on a surveyor. 8. Finishing and polishing with rotary instruments.

### 2.6 Operator preference

Operator preference was assessed with a questionnaire with a two‐choice question in a single selection mode: digital workflow or conventional workflow.

### 2.7 Difficulty and effectiveness

The perception of difficulty and effectiveness for both the digital and conventional workflow were assessed by a visual analogue scale (VAS). Participants were asked to draw a vertical line on a 100 mm non‐numeric line. For analysis, the answers were converted to a numerical value from 0 (“not difficult/effective”) to 100 (“very difficult/effective”) (Joda et al., 2017).

### 2.8 Operating time

**Figure 3:**
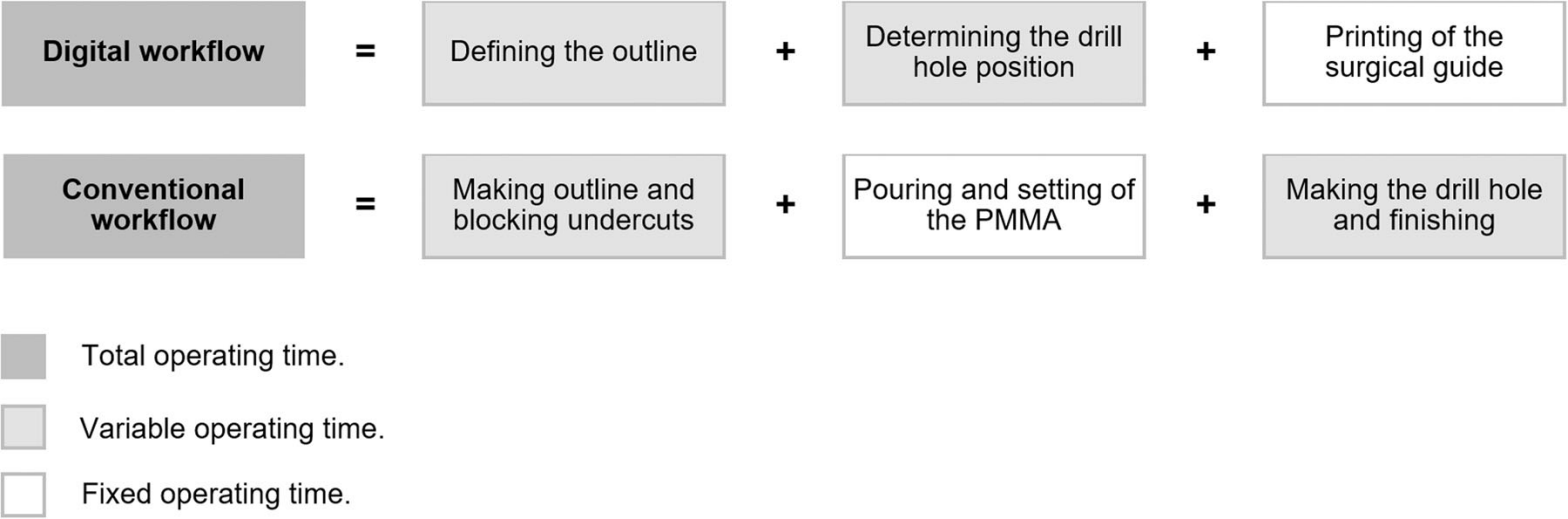
Total, variable and fixed operating time of the digital and conventional workflow.

Operating time for both fabrication methods was measured in minutes and seconds with a stopwatch by calibrated observers (KHH, VJJD). Both the digital and conventional workflows were divided into three steps (described in Table 1 and Table 2). Of these steps, two had a variable operating time, determined by the time it took the participant to complete the all the procedures of the workflow, and one had a fixed operating time, mainly determined by the techniques and materials used (e.g., print time). Total operating time was calculated by adding variable and fixed operating time, as shown in Figure 3.

### 2.9 Sample size

The variable perception of difficulty was used to estimate the number of participants needed. Difficulty was defined by the outcome of a questionnaire with a VAS (range 0–100). The difference between the means worth detecting was considered to be 10 points and the assumed standard deviation (SD) was 15 points (Joda et al., 2017). Thus, a total of 20 participants in each group were required to detect a statistically significant (α = .05) difference with 80% power (G*Power, Version 3.1, Heinrich Heine University, Dusseldorf, Germany).

### 2.10 Statistical analysis

The continuous data were checked for normality with the Shapiro–Wilk test. Categorical variables were assessed with contingency tables. Numerical variables were descriptively analyzed with sample means, SD, minimum and maximum values. Perceived difficulty, effectiveness and operating time were statistically analyzed by the Paired‐Samples T Test within the groups and by the Independent‐Samples T Test between the groups. The cut‐off point of *ɑ* = .05 was used to indicate statistical significance. All the statistical analyses were performed with a statistical software (IBM SPSS Statistics, Version 28.0, Armonk, NY, United States).

## 3 RESULTS

The participants’ mean age was 23.7 years (±0.8, range 23–25) for the students and 45.9 years (±12.9, range 26–64) for the dentists. Of the students, 65% were female and of the dentists, 75% were male. The dentists had a mean clinical experience of 20.1 years (±12.8, range 1–37). Each participant watched the tutorial video for both the digital and conventional workflow once and did not ask to watch either video again.

### 3.1 Operator preference

The digital workflow was preferred by 95% of the students and 70% of the dentists. Operator preference for the digital workflow totaled 82.5%.

### 3.2 Difficulty and effectiveness

When analyzing participants’ responses regarding perceived difficulty and effectiveness of the digital and conventional workflow, both groups tended to favor the digital workflow, except for the difficulty assessment in the dentist group. The scores for perceived difficulty and effectiveness did not differ significantly between the groups, except for difficulty for the conventional workflow (Table 3).

**Table 3:** Perception of difficulty and effectiveness (mean ± SD) for the digital and conventional workflow.

	Group	Digital	Conventional	*p* a
Difficulty	Students	29.1 ± 19.2	42.4 ± 20.1	.04
	Dentists	38.4 ± 25.8	22.0 ± 15.2	.007
	***p*** a	.205	<.001	
Effectiveness	Students	77.1 ± 10.3	60.1 ± 16.4	<.001
	Dentists	73.6 ± 21.9	64.1 ± 20.1	.103
	***p*** a	.527	.491	

*Note*: Mean VAS score from 0 (“not difficult/effective”) to 100 (“very difficult/effective”).

^a^ Paired‐Samples T Test within the groups, Independent‐Samples T Test between the groups. Statistical significance at *p* <.05.

### 3.3 Operating time

The variable operating time of both students and dentists was shorter for the digital workflow (Table 4). The students performed the procedures of the digital workflow faster than the dentists, while the dentists were faster than the students in the conventional workflow. The fixed operating time for the digital workflow (consisting of 3D printing time, removing excess print material and washing the surgical guide in ethanol) was 45 min after repeated measurements. For the conventional workflow, the fixed operating time (consisting of the pouring and polymerization time of the PMMA) was 26 min after repeated measurements. Thus, the total operating time (mm:ss) amounted 57:34 ± 2:24 (students) and 63:07 ± 6:03 (dentists) for the digital, and 48:20 ± 3:59 (students) and 46:16 ± 4:03 (dentists) for the conventional workflow.

**Table 4:** Operating time (mean ± SD) for the digital and conventional workflow.

	Group	Digital	Conventional	*p* a
Operating time	Students	12:34 ± 2:24	22:20 ± 3:59	<.001
	Dentists	18:07 ± 6:03	20:16 ± 4:03	.054
	***p*** a	<.001	.113	

*Note*: Operating time in minutes and seconds (mm:ss).

^a^ Paired‐Samples T Test within the groups, Independent‐Samples T Test between the groups. Statistical significance at *p* < .05.

## 4 DISCUSSION

In this randomized crossover study, the digital and conventional fabrication of a surgical guide on a standardized model with a single missing molar was evaluated among participants with varying levels of dental experience. The digital fabrication of surgical guides was perceived as less difficult and more effective than conventional fabrication by the students, but not by the dentists. Thus, the first and second hypotheses were partially rejected. Both students and dentists required less operating time for digital surgical guide fabrication, although the difference was only statistically significant for the students. Therefore the third hypothesis was partially rejected.

The operator preference was in favor of the digital workflow for students (95%) as well as dentists (70%) after using both techniques. For the students, this is in line with studies that examined operator preference towards digital and conventional impression making, reporting higher preference towards the digital workflow (Joda et al., 2017; Lee & Gallucci, 2013; Lee & Macarthur, & Gallucci, 2013; Zitzmann et al., 2017). That dentists also prefer the digital workflow over the conventional workflow for surgical guide fabrication is inconsistent with the literature comparing impression making techniques, probably because the dentists had prior experience with conventional impression making and no prior experience with surgical guide fabrication in the present study (Joda et al., 2017; Lee & Macarthur, & Gallucci, 2013).

The perceived difficulty of the digital workflow was relatively low (29.1 ± 9.2) among students, and significantly lower than the conventional workflow (42.4 ± 20.1). This indicates that students with no prior experience feel that the steps involved are not difficult and that they feel confident in achieving a consistent end result. These scores are similar to the perceived difficulty for digital impression making in two studies in which the participants were also dental students, reporting difficulty levels of 30.6 ± 17.6 (Lee & Macarthur, & Gallucci, 2013) and 29.7 ± 23.6 (Zitzmann et al., 2017) respectively. An interesting finding is that dentists found the digital fabrication method significantly more difficult, unlike the students. This may be explained by the fact that the students in this study were, on average, younger than the dentists and therefore possibly more accustomed to working with computer software.

The perceived effectiveness of both fabrication methods did not differ significantly between the groups. However, the students found the digital workflow (77.1 ± 10.3) significantly more effective than the conventional workflow (60.1 ± 16.4). All participants reported relatively high efficiency, indicating that they felt capable of producing the end result in a logical and timely manner. Additionally, the results show that the learning curve of manufacturing surgical guides is favorable after watching a tutorial video.

Both students and dentists were faster in surgical guide fabrication by using the digital workflow. That the digital workflow is faster, is consistent with another study comparing digital and conventional impression making (Zitzmann et al., 2017). In this study, we chose to compare only the variable operating time between the two manufacturing methods. The reason for this is that the fixed operating time is more material‐dependent than operator‐dependent. When the fixed operating time is added, the digital workflow takes more time due to the fact that the 3D printing time is longer than the polymerization time of PMMA in the conventional workflow. However, it can be argued that multiple surgical guides can be printed in one sequence, whereas the conventional workflow is limited to one surgical guide at a time due to the working time of PMMA.

The digital fabrication method for surgical guides described in this study has steps similar to the conventional workflow, so a clinically relevant comparison could be made. The results indicate that the digital fabrication method can be used in dental education programs. However, generalizability to the dental office is limited, as surgical guide fabrication is still often outsourced to a dental laboratory. Another limitation is that a blinded experiment cannot be conducted in the current study protocol. A possible factor is that the digital workflow is seen as more state‐of‐the‐art and thus a desirable response to the questionnaire. To get a full overview of how students experience the new technologies, future research is needed to combine digital impressions with other recently introduced features such as low‐noise instruments and computerized anesthesia devices (Kim et al., 2022; Vitale et al., 2023). The aim of the present study was to evaluate surgical guide fabrication. Therefore, creation of the set‐up was not part of the procedures for the participants. While it was shown that students also prefer a digital workflow for full‐contour crown fabrication on a training model, (Douglas et al., 2014) creation of the set‐up may be a more difficult part of the implant treatment planning workflow. Thus, statements can only be made about the part where the surgical guide is created. Further research could evaluate the digital and conventional creation of a set‐up as part of the complete workflow.

## 5 CONCLUSION

Within the limitations of this study, the following conclusions can be drawn. Both students and dentists prefer the digital workflow for surgical guide fabrication. Students perceive the digital workflow as less difficult and more effective than the conventional workflow. The operating time for fabricating a surgical guide is shorter with a digital workflow. The results of this study indicate that digital fabrication techniques for surgical guides can be incorporated into the dental curriculum to teach students about digital technologies in implant dentistry.

## AUTHOR CONTRIBUTIONS

V.J.J. Donker Conceptualization, Data Curation, Formal Analysis, Investigation, Methodology, Project Administration, Software, Visualization, Writing—Original Draft. K.H. Heijs Data Curation, Investigation. C.W.P. Pol Conceptualization, Methodology, Supervision, Writing—Review & Editing. H.J.A. Meijer Conceptualization, Methodology, Supervision, Project Administration, Writing—Review & Editing.

## CONFLICT OF INTEREST STATEMENT

The authors declare no conflict of interest.

## Data Availability

The data that support the findings of this study are available from the corresponding author, upon reasonable request.
